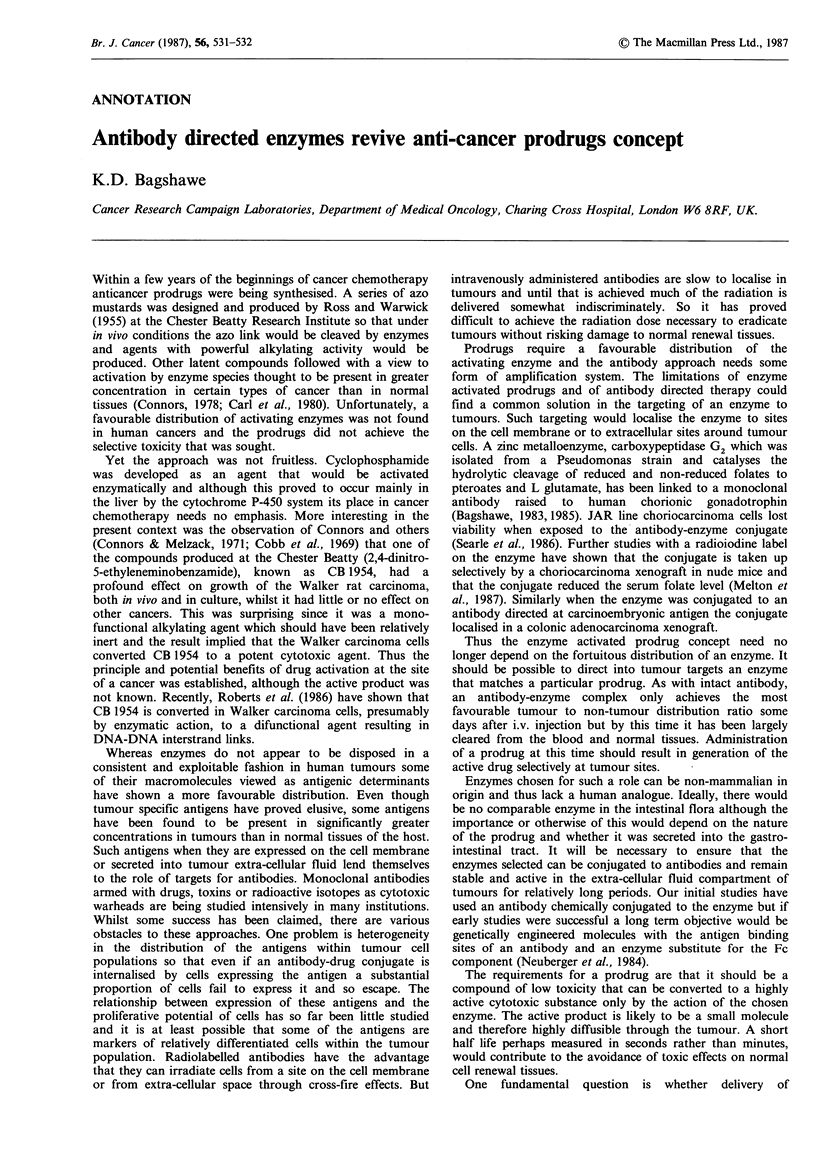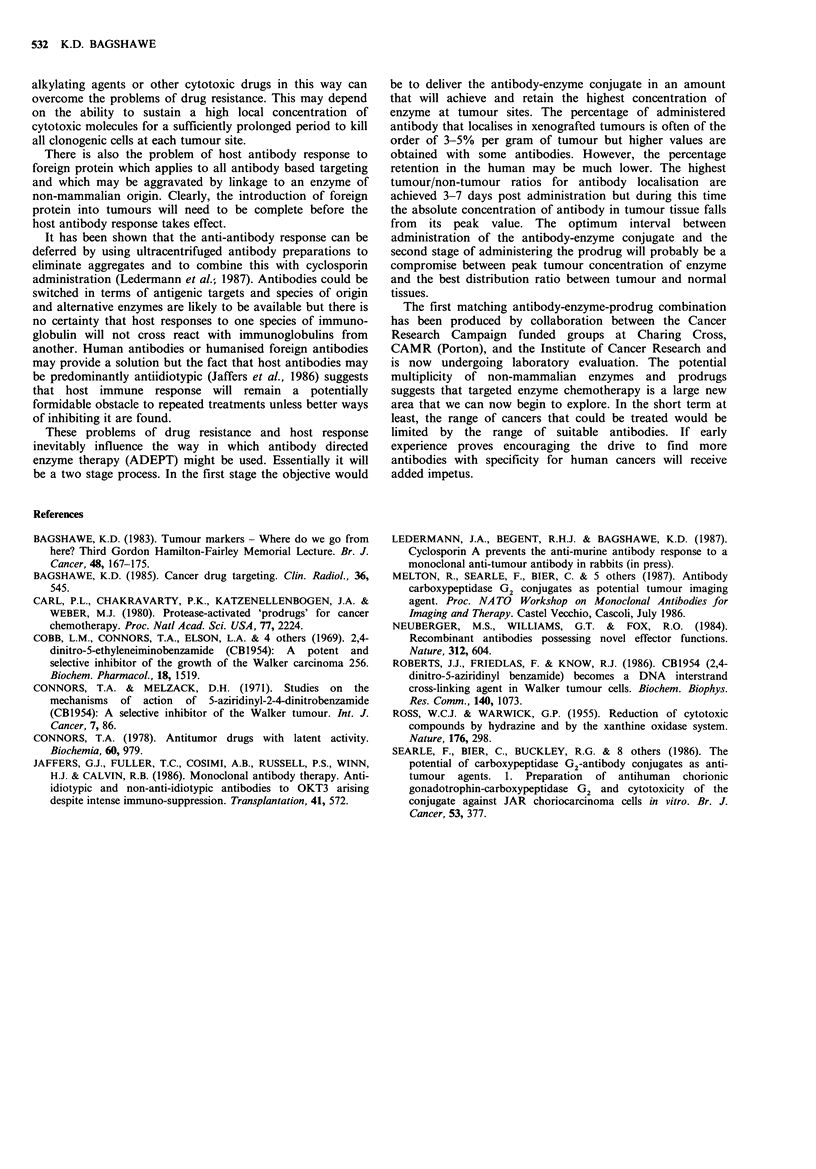# Antibody directed enzymes revive anti-cancer prodrugs concept.

**DOI:** 10.1038/bjc.1987.237

**Published:** 1987-11

**Authors:** K. D. Bagshawe

**Affiliations:** Department of Medical Oncology, Charing Cross Hospital, London, UK.


Br. J. Cancer (1987), 56, 531–532 © The Macmillan Press Ltd., 1987

ANNOTATION

### Antibody directed enzymes revive anti-cancer prodrugs concept

K.D. Bagshawe

*Cancer Research Campaign Laboratories, Department of Medical Oncology, Charing Cross Hospital, London W6 8RF, UK.*

Within a few years of the beginnings of cancer chemotherapy anticancer prodrugs were being synthesised. A series of azo mustards was designed and produced by Ross and Warwick (1955) at the Chester Beatty Research Institute so that under *in vivo* conditions the azo link would be cleaved by enzymes and agents with powerful alkylating activity would be produced. Other latent compounds followed with a view to activation by enzyme species thought to be present in greater concentration in certain types of cancer than in normal tissues (Connors, 1978; Carl *et al.*, 1980). Unfortunately, a favourable distribution of activating enzymes was not found in human cancers and the prodrugs did not achieve the selective toxicity that was sought.

Yet the approach was not fruitless. Cyclophosphamide was developed as an agent that would be activated enzymatically and although this proved to occur mainly in the liver by the cytochrome P-450 system its place in cancer chemotherapy needs no emphasis. More interesting in the present context was the observation of Connors and others (Connors & Melzack, 1971; Cobb *et al.*, 1969) that one of the compounds produced at the Chester Beatty (2,4-dinitro-5-ethyleneminobenzamide), known as CB 1954, had a profound effect on growth of the Walker rat carcinoma, both *in vivo* and in culture, whilst it had little or no effect on other cancers. This was surprising since it was a mono-functional alkylating agent which should have been relatively inert and the result implied that the Walker carcinoma cells converted CB 1954 to a potent cytotoxic agent. Thus the principle and potential benefits of drug activation at the site of a cancer was established, although the active product was not known. Recently, Roberts *et al.* (1986) have shown that CB 1954 is converted in Walker carcinoma cells, presumably by enzymatic action, to a difunctional agent resulting in DNA-DNA interstrand links.

Whereas enzymes do not appear to be disposed in a consistent and exploitable fashion in human tumours some of their macromolecules viewed as antigenic determinants have shown a more favourable distribution. Even though tumour specific antigens have proved elusive, some antigens have been found to be present in significantly greater concentrations in tumours than in normal tissues of the host. Such antigens when they are expressed on the cell membrane or secreted into tumour extra-cellular fluid lend themselves to the role of targets for antibodies. Monoclonal antibodies armed with drugs, toxins or radioactive isotopes as cytotoxic warheads are being studied intensively in many institutions. Whilst some success has been claimed, there are various obstacles to these approaches. One problem is heterogeneity in the distribution of the antigens within tumour cell populations so that even if an antibody-drug conjugate is internalised by cells expressing the antigen a substantial proportion of cells fail to express it and so escape. The relationship between expression of these antigens and the proliferative potential of cells has so far been little studied and it is at least possible that some of the antigens are markers of relatively differentiated cells within the tumour population. Radiolabelled antibodies have the advantage that they can irradiate cells from a site on the cell membrane or from extra-cellular space through cross-fire effects. But intravenously administered antibodies are slow to localise in tumours and until that is achieved much of the radiation is delivered somewhat indiscriminately. So it has proved difficult to achieve the radiation dose necessary to eradicate tumours without risking damage to normal renewal tissues.

Prodrugs require a favourable distribution of the activating enzyme and the antibody approach needs some form of amplification system. The limitations of enzyme activated prodrugs and of antibody directed therapy could find a common solution in the targeting of an enzyme to tumours. Such targeting would localise the enzyme to sites on the cell membrane or to extracellular sites around tumour cells. A zinc metalloenzyme, carboxypeptidase $G_2$ which was isolated from a Pseudomonas strain and catalyses the hydrolytic cleavage of reduced and non-reduced folates to pteroates and L glutamate, has been linked to a monoclonal antibody raised to human chorionic gonadotrophin (Bagshawe, 1983, 1985). JAR line choriocarcinoma cells lost viability when exposed to the antibody-enzyme conjugate (Searle *et al.*, 1986). Further studies with a radioiodine label on the enzyme have shown that the conjugate is taken up selectively by a choriocarcinoma xenograft in nude mice and that the conjugate reduced the serum folate level (Melton *et al.*, 1987). Similarly when the enzyme was conjugated to an antibody directed at carcinoembryonic antigen the conjugate localised in a colonic adenocarcinoma xenograft.

Thus the enzyme activated prodrug concept need no longer depend on the fortuitous distribution of an enzyme. It should be possible to direct into tumour targets an enzyme that matches a particular prodrug. As with intact antibody, an antibody-enzyme complex only achieves the most favourable tumour to non-tumour distribution ratio some days after i.v. injection but by this time it has been largely cleared from the blood and normal tissues. Administration of a prodrug at this time should result in generation of the active drug selectively at tumour sites.

Enzymes chosen for such a role can be non-mammalian in origin and thus lack a human analogue. Ideally, there would be no comparable enzyme in the intestinal flora although the importance or otherwise of this would depend on the nature of the prodrug and whether it was secreted into the gastro-intestinal tract. It will be necessary to ensure that the enzymes selected can be conjugated to antibodies and remain stable and active in the extra-cellular fluid compartment of tumours for relatively long periods. Our initial studies have used an antibody chemically conjugated to the enzyme but if early studies were successful a long term objective would be genetically engineered molecules with the antigen binding sites of an antibody and an enzyme substitute for the Fc component (Neuberger *et al.*, 1984).

The requirements for a prodrug are that it should be a compound of low toxicity that can be converted to a highly active cytotoxic substance only by the action of the chosen enzyme. The active product is likely to be a small molecule and therefore highly diffusible through the tumour. A short half life perhaps measured in seconds rather than minutes, would contribute to the avoidance of toxic effects on normal cell renewal tissues.

One fundamental question is whether delivery of alkylating agents or other cytotoxic drugs in this way can overcome the problems of drug resistance. This may depend on the ability to sustain a high local concentration of cytotoxic molecules for a sufficiently prolonged period to kill all clonogenic cells at each tumour site.

There is also the problem of host antibody response to foreign protein which applies to all antibody based targeting and which may be aggravated by linkage to an enzyme of non-mammalian origin. Clearly, the introduction of foreign protein into tumours will need to be complete before the host antibody response takes effect.

It has been shown that the anti-antibody response can be deferred by using ultracentrifuged antibody preparations to eliminate aggregates and to combine this with cyclosporin administration (Ledermann *et al.*; 1987). Antibodies could be switched in terms of antigenic targets and species of origin and alternative enzymes are likely to be available but there is no certainty that host responses to one species of immunoglobulin will not cross react with immunoglobulins from another. Human antibodies or humanised foreign antibodies may provide a solution but the fact that host antibodies may be predominantly antiidiotypic (Jaffers *et al.*, 1986) suggests that host immune response will remain a potentially formidable obstacle to repeated treatments unless better ways of inhibiting it are found.

These problems of drug resistance and host response inevitably influence the way in which antibody directed enzyme therapy (ADEPT) might be used. Essentially it will be a two stage process. In the first stage the objective would be to deliver the antibody-enzyme conjugate in an amount that will achieve and retain the highest concentration of enzyme at tumour sites. The percentage of administered antibody that localises in xenografted tumours is often of the order of 3–5% per gram of tumour but higher values are obtained with some antibodies. However, the percentage retention in the human may be much lower. The highest tumour/non-tumour ratios for antibody localisation are achieved 3–7 days post administration but during this time the absolute concentration of antibody in tumour tissue falls from its peak value. The optimum interval between administration of the antibody-enzyme conjugate and the second stage of administering the prodrug will probably be a compromise between peak tumour concentration of enzyme and the best distribution ratio between tumour and normal tissues.

The first matching antibody-enzyme-prodrug combination has been produced by collaboration between the Cancer Research Campaign funded groups at Charing Cross, CAMR (Porton), and the Institute of Cancer Research and is now undergoing laboratory evaluation. The potential multiplicity of non-mammalian enzymes and prodrugs suggests that targeted enzyme chemotherapy is a large new area that we can now begin to explore. In the short term at least, the range of cancers that could be treated would be limited by the range of suitable antibodies. If early experience proves encouraging the drive to find more antibodies with specificity for human cancers will receive added impetus.

### References

BAGSHAWE, K.D. (1983). Tumour markers – Where do we go from here? Third Gordon Hamilton-Fairley Memorial Lecture. *Br. J. Cancer*, **48**, 167–175.

BAGSHAWE, K.D. (1985). Cancer drug targeting. *Clin. Radiol.*, **36**, 545.

CARL, P.L., CHAKRAVARTY, P.K., KATZENELLENBOGEN, J.A. & WEBER, M.J. (1980). Protease-activated 'prodrugs' for cancer chemotherapy. *Proc. Natl Acad. Sci. USA*, **77**, 2224.

COBB, L.M., CONNORS, T.A., ELSON, L.A. & 4 others (1969). 2,4-dinitro-5-ethyleneiminobenzamide (CB1954): A potent and selective inhibitor of the growth of the Walker carcinoma 256. *Biochem. Pharmacol.*, **18**, 1519.

CONNORS, T.A. & MELZACK, D.H. (1971). Studies on the mechanisms of action of 5-aziridinyl-2-4-dinitrobenzamide (CB1954): A selective inhibitor of the Walker tumour. *Int. J. Cancer*, **7**, 86.

CONNORS, T.A. (1978). Antitumor drugs with latent activity. *Biochemia*, **60**, 979.

JAFFERS, G.J., FULLER, T.C., COSIMI, A.B., RUSSELL, P.S., WINN, H.J. & CALVIN, R.B. (1986). Monoclonal antibody therapy. Anti-idiotypic and non-anti-idiotypic antibodies to OKT3 arising despite intense immuno-suppression. *Transplantation*, **41**, 572.

LEDERMANN, J.A., BEGENT, R.H.J. & BAGSHAWE, K.D. (1987). Cyclosporin A prevents the anti-murine antibody response to a monoclonal anti-tumour antibody in rabbits (in press).

MELTON, R., SEARLE, F., BIER, C. & 5 others (1987). Antibody carboxypeptidase $G_2$ conjugates as potential tumour imaging agent. *Proc. NATO Workshop on Monoclonal Antibodies for Imaging and Therapy*. Castel Vecchio, Cascoli, July 1986.

NEUBERGER, M.S., WILLIAMS, G.T. & FOX, R.O. (1984). Recombinant antibodies possessing novel effector functions. *Nature*, **312**, 604.

ROBERTS, J.J., FRIEDLAS, F. & KNOW, R.J. (1986). CB1954 (2,4-dinitro-5-aziridinyl benzamide) becomes a DNA interstrand cross-linking agent in Walker tumour cells. *Biochem. Biophys. Res. Comm.*, **140**, 1073.

ROSS, W.C.J. & WARWICK, G.P. (1955). Reduction of cytotoxic compounds by hydrazine and by the xanthine oxidase system. *Nature*, **176**, 298.

SEARLE, F., BIER, C., BUCKLEY, R.G. & 8 others (1986). The potential of carboxypeptidase $G_2$-antibody conjugates as anti-tumour agents. 1. Preparation of antihuman chorionic gonadotrophin-carboxypeptidase $G_2$ and cytotoxicity of the conjugate against JAR choriocarcinoma cells *in vitro*. *Br. J. Cancer*, **53**, 377.